# Retraction Note: Novel magnetic organic–inorganic hybrids based on aromatic polyamides and ZnFe_2_O_4_ nanoparticles with biological activity

**DOI:** 10.1038/s41598-025-86732-2

**Published:** 2025-01-21

**Authors:** Reza Eivazzadeh-Keihan, Mostafa Ghafori Gorab, Hooman Aghamirza Moghim Aliabadi, Mohammad mahdavi, Ali Reza Akbarzadeh, Ali Maleki, Hossein Ghafuri

**Affiliations:** 1 Catalysts and Organic Synthesis Research Laboratory, Department of Chemistry, Iran University of Science and Technology, 16846-13114 Tehran, Iran; 2 Protein Chemistry Laboratory, Department of Medical Biotechnology, Biotechnology Research Center, Pasteur Institute of Iran, Tehran, Iran; 3 Advanced Chemistry Studies Lab, Department of Chemistry, K. N. Toosi University of Technology, Tehran, Iran; 4 Endocrinology and Metabolism Research Center, Endocrinology and Metabolism Clinical Sciences Institute, Tehran University of Medical Sciences, Tehran, Iran; 5 Department of Chemistry, Iran University of Science and Technology, 16846_13114 Tehran, Iran

Retraction of: *Scientific Reports* 10.1038/s41598-021-99842-4, published online 13 October 2021

Editors have retracted this Article.

After publication of this Article, concerns about the data were brought to the attention of the Editors. Specifically, the well images presented in Fig. 8 appear to have been previously published in an prior article with common authors^1^, where the data are presented differently. The Editors requested that the Authors provide full raw data and copies of their ethics approval and protocol for the use of blood samples drawn from a human donor, but the Authors were not able to do so. The Editors no longer have confidence in the reliability of the results and findings presented in this Article.

Reza Eivazzadeh-Keihan, Hooman Aghamirza Moghim Aliabadi, Ali Maleki, and Hossein Ghafuri disagree with this retraction. Mostafa Ghafori Gorab and Mohammad Mahdavi did not explicitly state whether they agree or disagree with this retraction. Ali Reza Akbarzadeh did not respond to correspondence from the Editors regarding this retraction.

## References

[1] Eivazzadeh-Keihan, R. et al. Synthesis and characterization of cellulose, β-cyclodextrin, silk fibroin-based hydrogel containing copper-doped cobalt ferrite nanospheres and exploration of its biocompatibility. *J Nanostruct Chem* **13**, 103–113. 10.1007/s40097-022-00481-6 (2023).

